# Uterine Cervical Angioleiomyoma Mimicking Squamous Cell Carcinoma

**DOI:** 10.3390/diagnostics13142370

**Published:** 2023-07-14

**Authors:** Jiwon Lee, Seoyeon Shin, Jin-Hwi Kim, Su Lim Lee, Yosep Chong, Kyung Jin Seo, Kwangil Yim

**Affiliations:** 1 College of Medicine, The Catholic University of Korea, Seoul 06591, Republic of Korea; iatiajwl@catholic.ac.kr (J.L.); kiradin@songeui.ac.kr (S.S.); 2 Department of Obstetrics and Gynecology, College of Medicine, The Catholic University of Korea, Seoul 06591, Republic of Korea; kjh@catholic.ac.kr; 3 Department of Radiology, College of Medicine, The Catholic University of Korea, Seoul 06591, Republic of Korea; radlsl@catholic.ac.kr; 4 Department of Hospital Pathology, College of Medicine, The Catholic University of Korea, Seoul 06591, Republic of Korea; ychong@catholic.ac.kr (Y.C.); ywacko@catholic.ac.kr (K.J.S.)

**Keywords:** uterine cervix, angioleiomyoma, magnetic resonance imaging, squamous cell carcinoma mimicker, menorrhagia

## Abstract

Angioleiomyoma, a rare variant of leiomyoma, is a benign tumor of mesenchymal origin. Angioleiomyomas of the female urogenital tract are extremely rare, with only six cases of uterine cervical angioleiomyoma previously reported in the literature. In this case study, we report on a 49-year-old female patient who presented with menorrhagia whose initial magnetic resonance imaging (MRI) findings suggested cervical squamous cell carcinoma (SCC). However, following the hysterectomy, histological examination confirmed the lesion to be angioleiomyoma. To the best of our knowledge, there have been no previously reported cases of angioleiomyomas presenting with MRI findings that are suggestive of uterine SCC. Recognizing that angioleiomyomas can mimic uterine malignancies on MRI may prove beneficial for future diagnostic and treatment strategies.

**Figure 1:**
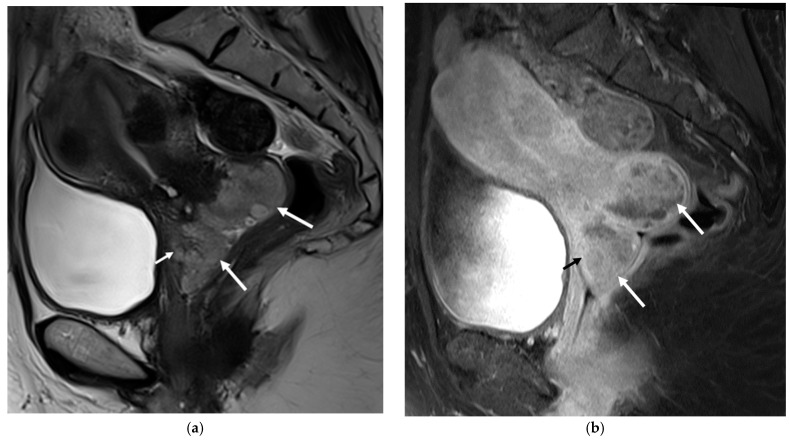
Magnetic resonance imaging (MRI) of the pelvis. Sagittal T2-weighted (**a**) and fat suppression T1-weighted (**b**) images revealed a lobulated hyperintense mass in the cervix (long arrows) protruding into the upper vagina, measuring 6.3 cm in size (anterior-posterior extension: 3.4 cm, width: 3.4 cm, oblique craniocaudal extension: 6.3 cm). Based on its large size, heterogenous enhancement, and probable vaginal infiltration observed on the images, this lesion was initially diagnosed as FIGO stage IIA cervical squamous cell carcinoma with an anterior fornix invasion (short arrow) [1]. Cervical cancer is a common malignancy with high morbidity and mortality [2]. Although magnetic resonance imaging (MRI) is useful for the detection and staging of cervical cancer, it is sometimes difficult to distinguish the cancerous lesion from benign or malignant mimickers based solely on MRI findings [3]. One of the cervical cancer mimickers that can be overlooked is angioleiomyoma of the uterine cervix. We present a case of angioleiomyoma of the cervix that was initially mistaken for cervical squamous cell carcinoma (SCC). A 49-year-old woman visited our outpatient clinic on account of uterine and cervical masses that were found on an outside CT scan. Her symptoms at presentation included heavy menstrual bleeding. An MRI examination was requested for a diagnostic work-up, and a cervical cancer-like lesion was discovered, as well as multiple intramural and subserosal uterine myomas (Figure 1). A Papanicolaou smear and punch biopsy were performed, and only low-grade squamous intraepithelial lesions (LSIL) were identified. The surgeon speculated that the lesion, due to its deep location within the endocervix, might not have been adequately biopsied and therefore proceeded with a diagnostic loop electrosurgical excision procedure (LEEP). By the LEEP specimen, the possibility of angioleiomyom was suggested, and LSIL was found.

**Figure 2:**
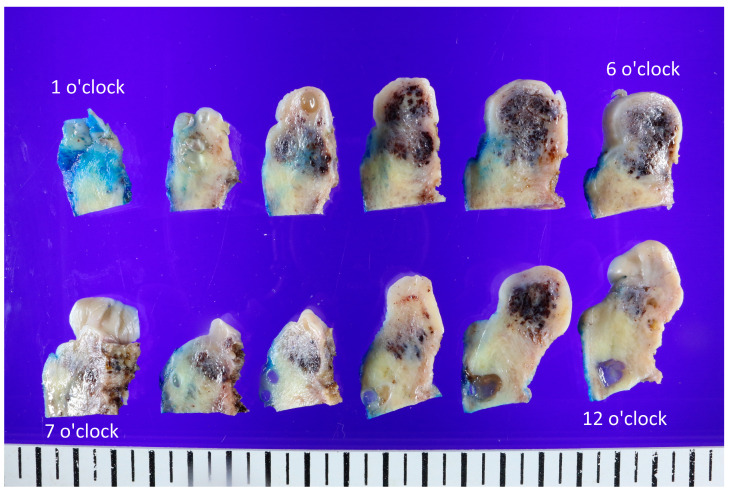
Representative gross findings of the uterine cervix. The whole uterus (9.5 cm × 5.6 cm × 4 cm, 158 g) was resected, and the cervix showed an ill-defined black-and-white heterogeneous mass (3.2 cm × 2.3 cm × 1.8 cm). The lesion was cut into different angles and observed. The cut surfaces were white and whorled, sometimes gray or pinkish, with focal regions of congested blood or hemorrhagic changes. However, based on the possibility of malignant potential, a robot-assisted single-site total laparoscopic hysterectomy with both salpingectomy and pelvic adhesiolysis was performed, and the whole uterus was extracted (Figure 2). The uterine cervix was noted for an ill-defined black to whitish heterogeneous mass (3.2 cm × 2.3 cm × 1.8 cm). The cut surfaces were white and whorled, sometimes gray or pinkish, with focal regions of congested blood or hemorrhages. This was in agreement with the macroscopic features of angioleiomyoma depicted in the literature, which has often been described as a well-circumscribed mass located in the submucosa, intramurally, or in the subserosa [4].

**Figure 3:**
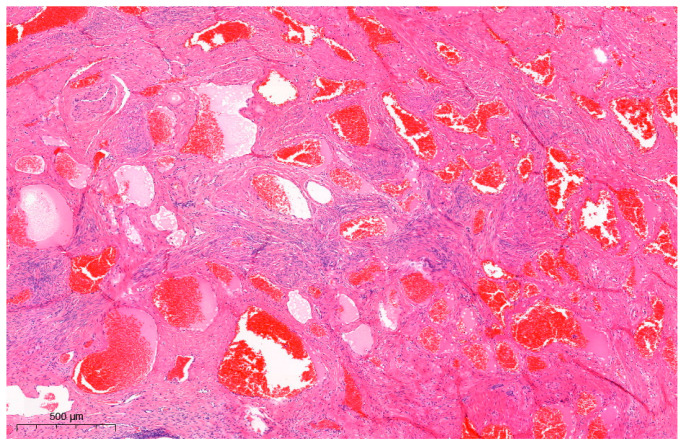
Microscopic findings of the uterine angioleiomyoma (H&E staining; ×50). Interlacing fascicles of spindle cells were seen, flanked by the proliferation of thick-walled blood vessels. Nuclear pleomorphism or mitotic figures were not observed. IHC results showed positivity for actin and desmin and negativity for CD-31, ERG, FLI-1, CD-34, and HMB-45 in stromal cells. The specimen was then viewed under a microscope (Figure 3). Hematoxylin and eosin (H & E) stained samples showed interlacing fascicles of spindle cells flanked by the proliferation of thick-walled blood vessels, with no signs of nuclear pleomorphism or mitotic figures. The immunohistochemistry (IHC) results were positive for actin and desmin and negative for CD-31, ERG, FLI-1, CD-34, and HMB-45 in stromal cells. This confirmed the lesion to be angioleiomyoma of the cervix: a benign tumor thought to be a variant of leiomyoma. The CARE (CAse REports) guidelines and checklist were used as a reference for the preparation of this report [5,6]. Angioleiomyoma is a rare variant of leiomyoma: a benign tumor of mesenchymal origin [4,7]. Angioleiomyoma of the female urogenital tract is extremely rare, with uterine cervical angioleiomyomas only being scantily reported. The following search strings were used to search the previous literature: “uterine cervix angioleiomyoma case” (5 results), “uterine cervical angioleiomyoma case” (10 results), and “uterine cervix AND angioleiomyoma” (6 results). After reviewing the content and overlapping searches, only six cases of angioleiomyoma of the uterine cervix were reported in the literature [8,9,10,11,12]; most of the angioleiomyomas were in the form of polyps. In the literature on uterine cervical angioleiomyoma, only one previous study by Hwang et al. [12] presented well-documented MR findings, which initially determined the tumor to be a cervical leiomyosarcoma. To our knowledge, this is the first reported case of cervical angioleiomyoma that initially presented as a cervical SCC mimicker. Unlike the case reported by Hwang et al. [12], which appeared as a heterogeneous cystic mass with hypointense nodular structures, our case presented as an infiltrative heterogeneous hyperintense lesion that was suggestive of a malignant process mimicking cervical SCC. In the case presented by Hwang et al. [12], the specimen showed a well-demarcated mass that contained many focal hemorrhagic components that were presumed to arise from the internal vascular structures, accompanied by cystic degeneration. The gross findings in our case also presented with dark hemorrhagic tissues, which were reflective of the predominant vascular component that appeared to encroach on normal tissue, in line with the MRI study showing vascular tissue that infiltrated into normal tissue, thereby mimicking a malignant process. Cervical cancer can appear as low signal intensity lesions on T1-weighted images and as intermediate to high signal intensity lesions on T2-weighted images with diffusion restriction (high signal intensity on diffusion-weighted imaging and low signal intensity on apparent diffusion coefficient maps) [3,13,14]. T2-weighted images without fat suppression are crucial for identifying the primary tumor and assessing its extent, which usually appears as an infiltrative or expansile mass with slight hyperintensity [13,15]. Some of the mimickers of cervical cancer include inflammatory processes such as infection (Actinomyces [16], Mycobacterium [17], Aspergillus [18], Klebsiella [19], Treponema [20]) and abscesses [21] or benign (malakoplakia [22], cystic glandular change [23,24], endometriosis [25], Nabothian cysts [23], and cervical polyps) and malignant (lymphoma, leukemia [26], solid tumor [27,28], metastatic lesions) processes [3]. On an MRI, these lesions often present as heterogeneous intermediate-to-high-signal-intensity lesions on T2-weighted images with diffusion restriction, similar to cervical cancer. The radiological appearance of angioleiomyomas, as reported in the literature, is nonspecific [12]. Typically, angioleiomyomas are described as well-defined, single, and unilocular. However, they can sometimes present as a single unilocular necrotic mass accompanied by an internal solid or laminated form [12], and cystic degeneration can manifest in larger-sized angioleiomyomas [29], mimicking a malignant process. Biopsies and histopathological analyses are necessary when a diagnosis is difficult to confirm [30]. Microscopically, angioleiomyoma appears as interlacing fascicles of spindle cells surrounding abundant thick-walled blood vessels. Malignant features such as pleomorphism, mitosis, hyperchromatic nuclei, or necrosis are not seen [4,12]. Angioleiomyoma is known to stain positively with smooth muscle actin, desmin, vimentin, and h-caldesmon while staining negatively with HMB-45 and MART-1 [4]. Morphological mimicking encompasses angiomyofibroblastoma (demonstrating ER, PR, and CD-34 positivity) [31,32], perivascular epithelioid cell neoplasm (exhibiting HMB-45 positivity) [4], and hemangioendothelioma (expressing CD-31, ERG, and FLI-1 positivity) [33,34]. These differentials were excluded in our case based on the IHC findings. The angioleiomyoma specimen in the previous case by Hwang et al. that mimicked malignancy was reported to show numerable thick-walled vessels embedded in the smooth muscle cells, accounting for its resemblance to leiomyosarcoma. Similarly, our specimen also contained abundant vascular components that correlated with the infiltration-like lesion seen on the imaging. Based on these findings, we suggest that angioleiomyomas with a predominant vascular component can mimic cervical malignancy and should be considered while evaluating patients that present with a cervical mass. To summarize, cervical cancer is the second most common malignancy of the female genital tract in Western countries and the third most common cause of cancer-related deaths in females in developing countries [3,15]. Although imaging modalities like MRI can accurately assess its prognostic indicators, including tumor size, parametrial invasion, pelvic sidewall invasion, and lymph node invasion, some pitfalls make it difficult when differentiating cancer from cervical cancer mimickers [3]. We reported a case of a cervical mass that was initially suspected to be cervical malignancy but was later confirmed to be angioleiomyoma on histopathological examination. This is the first reported case of cervical angioleiomyoma with a full histological and radiological profile that was initially mistaken for cervical SCC. The predominant vascular component observed on pathology presumably led to the irregular distribution of the margin of the mass as observed in the MRI studies. We propose that angioleiomyomas with a predominant vascular component can act as a cervical cancer mimicker and should be included in the differentials when assessing a cervical mass. As preoperative diagnosis based on MRI is not always reliable, the pitfalls of image-based diagnosis should be considered when treating patients with cervical masses.

## Data Availability

The data presented in this study are available upon reasonable request from the corresponding author.
